# Development of an mHealth trauma registry in the Middle East using an implementation science framework

**DOI:** 10.1080/16549716.2017.1380360

**Published:** 2017-10-13

**Authors:** Amber Mehmood, Edward Chan, Katharine Allen, Ammar Al-Kashmiri, Ali Al-Busaidi, Jehan Al-Abri, Mohamed Al-Yazidi, Abdullah Al-Maniri, Adnan A. Hyder

**Affiliations:** ^a^ Johns Hopkins International Injury Research Unit, Department of International Health, Johns Hopkins Bloomberg School of Public Health, Baltimore, MD, USA; ^b^ Ministry of Health, Muscat, Oman; ^c^ The Research Council of Oman, Muscat, Oman

**Keywords:** Trauma registry, injury surveillance, mHealth, implementation framework, Oman, Middle East

## Abstract

**Background**: Trauma registries (TRs) play a vital role in the assessment of trauma care, but are often underutilized in countries with a high burden of injuries.

**Objectives**: We investigated whether information and communications technology (ICT) such as mobile health (mHealth) could enable the design of a tablet-based application for healthcare professionals. This would be used to inform trauma care and acquire surveillance data for injury control and prevention in Oman. This paper focuses on documenting the implementation process in a healthcare setting.

**Methods**: The study was conducted using an ICT implementation framework consisting of multistep assessment, development and pilot testing of an electronic tablet-based TR. The pilot study was conducted at two large hospitals in Oman, followed by detailed evaluation of the process, system and impact of implementation.

**Results**: The registry was designed to provide comprehensive information on each trauma case from the location of injury until hospital discharge, with variables organized to cover 11 domains of demographic and clinical information. The pilot study demonstrated that the registry was user friendly and reliable, and the implementation framework was useful in planning for the Omani hospital setting. Data collection by trained and dedicated nurses proved to be more feasible, efficient and reliable than real-time data entry by care providers.

**Conclusions**: The initial results show the promising potential of a user-friendly, comprehensive electronic TR through the use of mHealth tools. The pilot test in two hospitals indicates that the registry can be used to create a multicenter trauma database.

## Background

Injuries are the leading cause of years of life lost and disability-adjusted life-years in Oman [1,2], where the age-standardized mortality due to injuries is 53 per 100,000 population. Injuries place a considerable burden on the healthcare system; in 2013, injuries were recorded at a rate of 877 per 10,000 population across all Ministry of Health (MOH) institutions [3]. Middle Eastern countries such as Oman, where the injury burden is high, often face a lack of reliable and up-to-date information on injury epidemiology and care [4,5]. Despite being equipped with good health information systems and electronic medical records (EMRs), a lack of in-depth data on the quality of trauma care and effectiveness of injury prevention initiatives limits the ability of clinicians and public health practitioners to develop focused interventions for injuries [6]. Given this challenge, rapidly growing economies with a high burden of injuries need efficient ways to collect such evidence instead of relying on annual reports of routine administrative data with limited clinical information [7,8]. For clinicians, researchers and policymakers alike, the need for an evidence base for performance improvement and evaluation of trauma care practice has been identified [9].

Trauma registries (TRs) are electronic databases used in many high-income countries to monitor and enhance the quality of trauma care and provide an evidence base for injury control and policy development [6]. Registries differ from EMRs by virtue of clear case definitions and a focus on recording clinical information around trauma care [10,11]. Unlike injury surveillance systems, TRs have a dual function of assessing the quality of clinical care and providing injury surveillance data. The assessment of clinical performance and the development of effective injury control strategies require the systematic acquisition and analysis of data [12]. TRs have been shown to play an integral role in this complex framework [6]. Commercially available TRs are suitable for highly developed health management information systems, but the depth and breadth of information collected by such registries may be redundant or irrelevant in other countries [13]. Currently, Oman does not have a functional TR in any of the hospitals run by the MOH, which is the main provider of trauma care services across the country [3,14].

With the emergence of modern information and communications technology (ICT), and the extensive use of mobile phones and other wireless communication devices, mobile health (mHealth) has a great potential to support public health and clinical practice [15,16]. A common mHealth framework could be applied to improve trauma care systems and injury control through the use of point-of-care diagnosis, applications for awareness and behavioral change, TRs, electronic health records, decision support systems, provider-to-provider communications, training and education [17]. ICT use in healthcare settings is influenced by many factors and thus the interventions for successful implementation must be multidimensional [18].

We postulated that mHealth tools could enable the design of a TR and capture surveillance data for injury control and prevention. Moreover, using a framework for program planning that is grounded in behavioral change theory could potentially facilitate the uptake of the mHealth tool [19]. The overall goal of this study was the development of an mHealth platform that would become the precursor to a multicenter trauma database to inform trauma care efforts in Oman. The specific objectives of this project were: (1) to define the essential constituents of a data set useful for injury control and performance improvement in trauma care; (2) to identify and develop an appropriate mHealth platform for data collection; and (3) to pilot-test the implementation of a registry using an ICT implementation framework, to understand and facilitate the usage of the mHealth platform. In this article, we describe the process of developing and pilot-testing an mHealth-based TR application in Oman.

## ICT implementation framework

**Figure 1.**
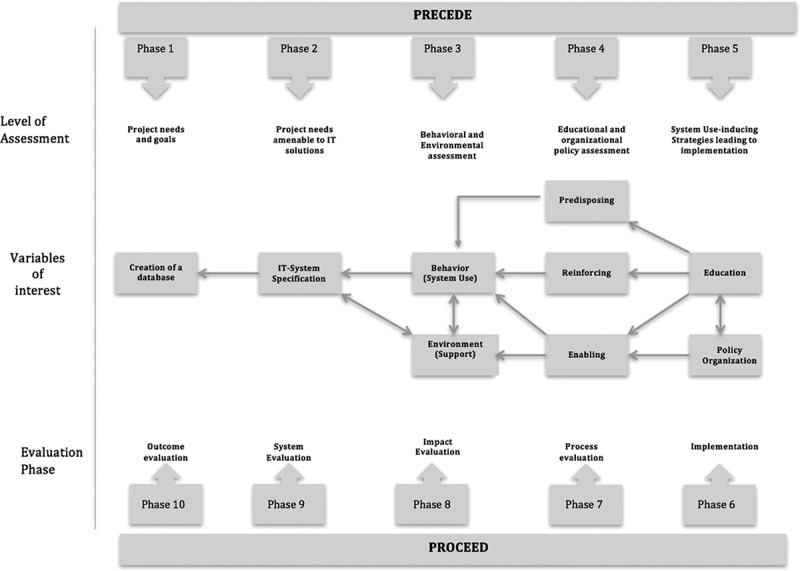
Information and communications technology implementation framework based on the Precede–Proceed model. (Adapted from Kukafka et al., 2003 [18]).

For this study in Oman, we adopted a modified ICT implementation framework illustrated by Kukafka et al. [18]. The framework is based on the Precede–Proceed model initially described in the context of behavioral change theories [20,21]. The ‘Precede’ or assessment phase of the framework consists of several steps that help in choosing a technology that matches the needs of the project and expectations of end-users. Important activities in this phase include engaging with different stakeholders to outline goals, understand their perspectives and learn about organizational capacity. Specific components of the needs and objectives that could be managed by an information system help to define the specifications and functionality of the proposed mHealth system. Behavioral and environmental assessment entails a step-by-step systematic analysis to identify behaviors that need to be performed (by end-users such as nurses, doctors and data-collection teams) to make sure that a particular system is used. Environmental factors identified at this stage have the potential to influence ICT use and may interact at a physical (e.g. mobile phone availability, access to Wi-Fi) or social (e.g. acceptability of smartphone or mobile devices in the work environment) level. This level of specificity makes it possible to recognize tangible behavioral aspects where individually focused behavioral strategies, e.g. skills training, could be supplemented by environmental support, e.g. improving Wi-Fi access (Figure 1).

Educational and organizational policy assessment helps in identifying predisposing factors such as cognitive dimensions, self-efficacy to perform the tasks, and the perceived usefulness of the system and its related activities. Likewise, enabling factors of the organization such as available resources, supportive policies, and accessibility to the information technology (IT) system are enhanced by reinforcing factors such as incentives, remuneration, positive recognition and rewards to motivate people to continue to use the system. Finally, developing and implementing approaches that are specifically targeted at influencing the aforementioned factors through education and favorable policy change help in effective planning of the program, which leads to the ‘Proceed’ phase, with implementation and evaluation of the program. The assessment process helps to identify the variables of interest or indicators that could be used in the evaluation of the implementation process, impact, system and outcomes of the program. Effective education and organizational policies are the foundation of a cascade of actions directed towards changing behavior by imparting cognitive skills, creating an enabling environment and reinforcing positive behavior, and thus achieving the goals of the project (Figure 1).

We applied this modified ICT framework in a healthcare setting, studied various dimensions of project and organizational needs with the IT solutions, and integrated them with implementation strategies. In this paper, we will report on the development and implementation processes of the mHealth registry according to this framework.

### Precede phase

The Precede phase of our project entailed various stages of assessment and planning, including: (1) defining the objectives and scope of the TR through a task force that corresponded with the project’s needs and goals; (2) exploring potential ICT platforms to fulfill the project’s needs and goals; (3) understanding the behavioral and environmental factors; (4) outlining organizational resources for the implementation of the electronic TR; and (5) developing strategies to facilitate the use of the IT platform. These steps are described in detail below.

#### Project needs and goals

As a first step, a multidisciplinary task force was created in Oman to define project goals and needs. The task force included public health researchers, health IT experts, emergency physicians and trauma surgeons. Several strategies were employed for a thorough assessment, including the examination of available electronic data sources, review of 1000 charts of consecutive trauma patients to understand the information captured through routine medical records, review of injury surveillance tools, and engagement with a number of stakeholders in government and academic institutions. Several meetings with the MOH IT department were held to explore the EMR, called Al-Shifa, and possible integration of the TR with the Al-Shifa system. During the assessment, the following observations were made. Oman has a good EMR system in all hospitals with distinguishable injury cases, but there is s lack of granularity in the data on the burden and care of injured patients [14]. This problem could potentially be addressed by a comprehensive tool by embedding the indicators and outcomes pertinent to injury control, access to care, quality of care and outcome of trauma patients admitted to Omani hospitals. Special emphasis was put on the recommendations of the World Health Organization’s injury surveillance guidelines, as well as the American College of Surgeons Committee on Trauma [22,23].

#### Project needs amenable to mHealth solutions

Given the overall project goal, there was a need for the proof of concept that an electronic TR for could be developed and implemented, before making it available in all Omani hospitals through EMRs. For this purpose, an mHealth platform was developed and pretested. Several steps were taken to develop a user-friendly and standardized data-collection tool, as outlined below.

For this project, Open Data Kit (ODK; https://opendatakit.org/) and other open source technologies were used to manage mobile data collection. First, a local secure sockets layer (SSL) secured Formhub (http://formhub.org) and Linux server was installed. Secondly, questionnaires were created through XLSFORM using Microsoft Excel and uploaded into the local Formhub, through a secure Internet connection. Thirdly, once the questionnaire had been pretested and finalized, the form was downloaded to Android tablets using the ODK app. This platform has been tested and recommended owing to its simplicity, easy deployment on Android devices and ability to create a multiple-user database for large data aggregation, sharing and visualization. Once data collection for individual trauma cases was complete, the data could be uploaded to a server with 2048-bit SSL encryption.

#### Behavioral and environmental factors

After conducting several interviews and discussions with the staff and administrators at the participating hospitals, scoping visits to the hospitals and mapping trauma patient flow, several observations were noted. Omani hospitals are equipped with the Al-Shifa EMR system, which is uniform in structure and functions in all hospitals. All the staff in the hospitals are well versed in electronic data entry, and also familiar with international disease coding systems such as the International Classification of Diseases, 10th Revision – Clinical Modification (ICD-10-CM). The Al-Shifa system requires mostly free text entry and trauma-related details are not recorded in a standardized format. There was a perceived lack of retrievable information on trauma patients owing to the non-standardized format of documentation. Smartphone use was widespread, but Wi-Fi access was not optimal.

#### Educational and organizational policy factors

Various levels of healthcare providers, leading surgeons and hospital administrators were approached to find out about the care and referral system of the injured patients, and trauma data-sharing policies. In addition, key-informant interviews were conducted with the leadership of six referral hospitals, including administrators, staff and trauma care providers. Focus group discussions with providers in the emergency and inpatient departments provided in-depth information about the dynamics of injury-related data collection, including barriers, strengths and weaknesses of the EMR, availability of and access to fixed and mobile electronic devices, and organizational support in capacity development for various cadres of care providers. Many participants expressed the need for an injury surveillance system and better documentation of the clinical course of injured patients.

#### System use-inducing strategies

At the start of the pilot study, nurses from both hospitals were selected and trained in data collection; the selection was based on their day-to-day involvement in trauma care, their willingness to participate in the research project, and their eagerness to learn new knowledge and practice new skills. A small financial incentive was also added to the standard salary to encourage participation and support the nurses’ work. A week-long induction period included the training workshop, hands-on practice and real-time data entry. Three-day training workshops included fundamental elements of TRs, injury data abstraction from medical charts, data entry on a mobile device, injury coding such as e-codes, ICD-10-CM, and basic principles for various scaling and scoring systems including the Revised Trauma Score (RTS) and Injury Severity Score (ISS) [24,25]. The workshop was formatted to include lectures, workbooks, case scenarios, quizzes, online injury scoring calculators, and demonstrations to learn and practice new concepts.

All the participants were provided with individual handheld android devices and the TR user guide and appendices. The user guide was developed to outline the step-by-step details of data collection, and to provide a quick reference for ICD codes and ISS calculation. Classroom training was followed by practical sessions on real-time data entry in hospital under supervision, during which the Johns Hopkins International Injury Research Unit (JH-IIRU) team provided instant responses to queries such as potential cases and clarification on inclusion criteria, and feedback on their knowledge and skills. Before starting data collection, a group email and chat on WhatsApp were created for the data collectors to submit queries, and to ensure effective and efficient communication. Two team members from JH-IIRU monitored these accounts and responded promptly to any queries submitted by the data-collection teams.

**Figure 2.**
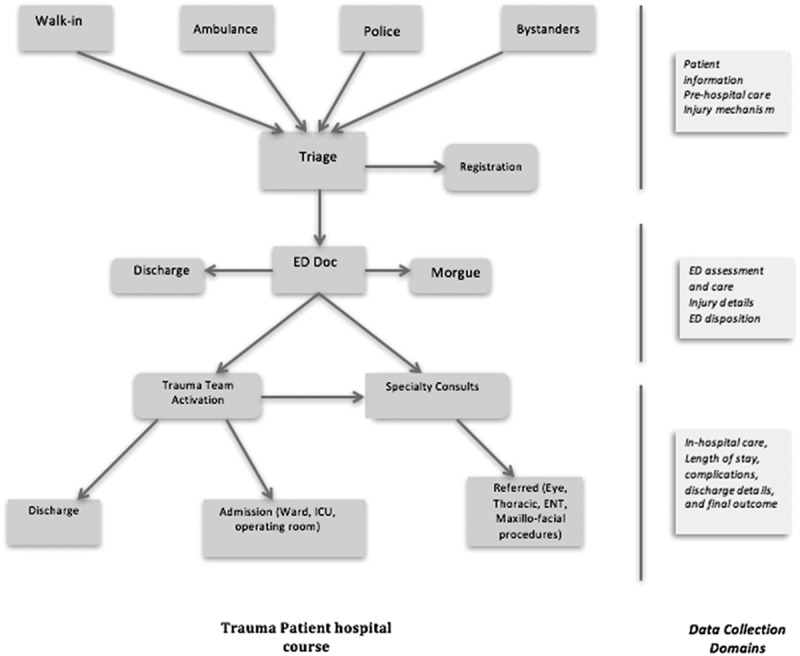
Typical course of a trauma patient and organization of data-collection domains. ED Doc, emergency department doctor; ICU, intensive care unit; ENT, ear, nose and throat.

Considering the busy hospital environment, particular attention was paid to making the interface user friendly to facilitate the data-entry process. The integration of smart checks to ensure completeness of data also minimized incorrect clinical information. Examples of such strategies included a predefined range of vital signs, skip patterns and mandatory fields. The variables were also organized after closely monitoring the healthcare providers’ workflow and the typical course of trauma patients at the study sites (Figure 2).

The strength of the Wi-Fi signal in different parts of the hospital and the availability of mobile data plans were important considerations in the selection of mobile devices for data collection. The ability to upload data to a secure server hosting the TR database was facilitated by the use of Android mobile devices with both Wi-Fi connection and SIM-card-supported data plans.

### Proceed phase

Since this paper focuses on the planning and pilot implementation of the mHealth registry, the implementation procedures, process, impact and system evaluations are described in detail.

#### Implementation of the registry

The Precede phase was followed by pilot implementation of this mHealth-based International Injury Research Unit trauma registry (IIRU-TR). Two sites in Oman were selected for the pilot implementation of the TR: Khoula Hospital and Nizwa Hospital. Khoula Hospital, located in Muscat, serves as a major referral center for trauma and burns. Khoula also serves multiple governorates outside Muscat and has a highly equipped emergency room with trauma resuscitation bays, a 24/7 multidisciplinary trauma team, and intensive care units and emergency theaters available. The hospital serves as the national Burns and Plastics Referral Center, as well as the leading neurosurgical facility. Nizwa Hospital is a secondary care hospital in the governorate of Ad-Dakhiliyah. It is located near an industrial area and receives patients not only from Ad-Dakhiliyah, but also from neighboring governorates. There are trained emergency physicians and trauma teams, but many severely injured trauma patients and those with traumatic brain injuries need to be transferred to the tertiary care centers of Muscat. Both hospitals conduct regular educational activities around trauma care, such as seminars and quality of care audits.

Pilot implementation was conducted over a 6 month period and all trauma patients who were admitted or had trauma team activation in the emergency room were included in the study, including emergency department deaths and dead-on-arrival cases. Ethical approval was obtained from the Omani MOH and the Johns Hopkins Bloomberg School of Public Health before the start of data collection. A stakeholders’ meeting was conducted to provide information about the project’s goals and objectives, details of the mHealth registry and the implementation plan. The stakeholders included hospital administrators, representative clinicians, nurses and MOH officials, and their feedback was used to recruit the data collectors. The process of implementation was monitored closely and several quality checks were put in place, which are described in subsequent sections.

#### Process evaluation

In the IIRU-TR, data were collected for each new case, edited and saved on the mobile device. A unique identifier (four-digit serial number) was assigned to each case instead of collecting personal identifiers. Once the case had been uploaded to the server, it was erased automatically from the tablet. For any further modification, the data collectors were supposed to notify the supervisors, who would keep a log of the changes and edit the record on the database.

Supervisors randomly selected 10% of cases for quality checks at both sites every month. In addition, four separate visits were made by the JH-IIRU team to both hospitals during the study period to cross-check the data, in order to ensure completeness and accuracy of selected variables such as vital signs, ICD codes, radiological reporting, time of arrival and length of stay. The data collectors shared monthly reports including the number of captured cases, active (still admitted) cases and completed entries.

Data capture was complete and secure data transfer to the server was successful in all cases. However, it was noticed that the Wi-Fi signals were poor in certain areas of both hospitals and thus data transfer of completed cases in bulk was more feasible using either Wi-Fi or a mobile data plan. As a result of quality checks, 194 errors (7.4%) were documented out of all captured cases. Examples include unknown arrival time, unknown length of stay in the emergency department and duplicate disposition errors. Of these errors, 71% were corrected after cross-checking. Only 12 cases were recorded as duplicates and were thus removed from the database. Data collectors sought guidance in finalizing ICD and Abbreviated Injury Scaling (AIS) codes in 32 cases (1.25%).

#### Impact evaluation of IIRU-TR use

The main impact of IIRU-TR was the establishment of a comprehensive database of more than 2500 eligible trauma cases with standardized and retrievable information. The performance of data collectors was measured by the frequency of completed forms uploaded on the server, the proportion of actual versus captured cases, and errors found on quality checks of the data. A follow-up group discussion with the data collectors and local supervisors was conducted at the end of the project to understand the utility, challenges, barriers and potential solutions. The majority reported that they would like to continue with the same tool and to attend regular refresher workshops, and identified the WhatsApp chat group as a useful support system to facilitate communication and as a source of learning with respect to difficult cases.

At the hospital level, the IIRU-TR implementation helped to inform the administrators about crucial trauma care processes. Examples include delays in the emergency department, the proportion of direct admissions and transfer cases, and emergency department deaths. The identification of these cases led to initiatives around quality improvement, preventable death audits and safe transfers between the hospitals.

#### System evaluation: TR specifications and database

IIRU-TR represented a system of injury data collection and storage that was comprehensive and user friendly. The variables were organized into 11 domains corresponding to different stages of the patients’ course after injury (Table 1). These variables capture basic injury surveillance information, such as sociodemographic information, external causes of injuries, place and location of the incident, and ICD-10 injury codes; risk factors, such as the use of drugs or alcohol, safety equipment, activity at the time of the incident and road user information; clinical details, such as triage code, ISS, prehospital and hospital assessment and management; variables related to quality of care, including prehospital time, length of stay in the emergency department, trauma team activation and complications; and outcome variables, such as survival, discharge and short-term disability.

**Table 1.** Domains of data collection and related variables.

	Domain	Variables
1	General patient information	Date and time of arrival
		Date and time of injury
		Age and gender
		Nationality
		Occupation and education
2	Prehospital care	Mode of arrival
		Care provided at the scene of injury
		Prehospital care provider
3	Prehospital assessment	Prehospital vital signs
		Ambulance triage code
4	Injury details	Location and place of occurrence
		Mechanism of injury
		External causes of injury
		Injured road user
		Safety equipment
5	ED assessment	ED vital signs
		Trauma team activation
		ED investigations
		Confirmed or suspected alcohol/substance use
6	ED treatment	ED treatment (meds, airway control, thoracostomy, CPR, etc.)
		Blood transfusion
7	ED disposition	Admitted
		Discharged (expired, sent home, AMA, referred)
		Time of exit from ED, ED length of stay
8	ICD-10 injury codes	ICD-10 injury codes
9	Inpatient care	Blood products
		ICU length of stay
		Operative procedures
		Complications
10	Injury Severity Score	Head and neck
		Face
		Chest thorax spine
		Abdomen pelvis lumbar
		Extremities
		External
11	Discharge details	Final disposition
		Date and time of discharge, overall length of stay

ED, emergency department; ICD-10, International Classification of Diseases, 10th revision; CPR, cardiopulmonary resuscitation; AMA, against medical advice; ICU, intensive care unit.

An important improvisation was the use of simplified descriptors for AIS codes, to help data collectors to assign an injury severity for each injury. This was done by merging nine AIS chapters into six groups of injury severity and carefully selecting the most common descriptors covering a range of severity scales in each region of the body [26]. The database was standardized and errors were minimized using various strategies: by exclusive use of drop-down menus and check boxes, minimizing free text entry, and using standardized definitions and injury details. The database was easily downloadable in Excel spreadsheet format and the fidelity of the system was maintained throughout the study period. No modifications were made to the questionnaire, data-collection process or server configurations.

### Outcome evaluation

Over the pilot period, data were collected on more than 2500 trauma cases; however, the detail of these cases is the subject of another manuscript. The TR developed for this project had features that made it easy to adopt and use. Simultaneous pilot testing of IIRU-TR in two hospitals demonstrated its potential to become a multicenter database. At the end of the pilot implementation, the JH-IIRU team held discussions with the MOH-IT department to plan the integration of the mHealth registry with the Al-Shifa system. Finally, another 3 day ‘train-the-trainer workshop’ was organized for the team of data collectors to help them to take a leading role in training future data collectors, and to provide instructions on the use of data to produce summary tables and reports for trauma care providers and hospital administrators.

## Discussion

Mobile technologies have opened up new avenues for global public health, in both developed and developing countries, owing to their ubiquitous availability, affordability, and ease of electronic data collection and transfer [17,27]. A successful mHealth tool could be applied to strengthen health systems by creating real-time data repositories such as TRs. These influence the development of the trauma system and encourage continuous quality improvement through communication and behavioral change, provider training and education, and utilization of clinical information at the individual and population levels.

The pilot test of mHealth-based IIRU-TR was facilitated using an implementation science framework, as well as implementation strategies also described by other investigators [28]. This framework helped in the identification of critically important enabling (knowledge and skills required for electronic data entry), predisposing (personal mobile device) and reinforcing factors (reporting mechanisms and instant feedback). These factors played an important role in tailoring strategies according to the environment, individual behavior and organizational policies. In addition, several implementation strategies were employed in this project, as summarized in Table 2. A comprehensive planning phase, with needs assessment, stakeholders’ engagement, and setting goals and objectives, was paired with scoping visits to gather information as well as identify opportunities and challenges, and was critical in selecting context-specific approaches and building relationships and long-term partnerships. Buy-in from the stakeholders was an important factor in the successful implementation, as previously highlighted by other researchers [12,29,30]. Strategies were directed towards developing the most feasible and user-friendly mHealth platform possible, as well as providing appropriate educational and training opportunities to use and adapt to this platform.

**Table 2.** Summary of implementation strategies used in the International Injury Research Unit trauma registry (IIRU-TR).

Planning	Conduct local needs assessment
	Build buy-in with stakeholders
	Consensus discussions with local stakeholders and experts
	Site visits
	Assess readiness, identify barriers
	Select strategies according to context
	Create academic partnership
	Develop relationships
Education	Conduct educational meetings
	Conduct specific training
	Develop effective material
	Inform and influence stakeholders
	Work with educational institutions
	Ongoing consultation
Restructuring	Revise roles (nurses as primary data collectors)
	Acquire new equipment (handheld devices, setting up server)
Quality management	Organize and conduct implementation team meetings
	Centralize technical assistance
	Develop system and associated tools
	Use data experts
	Data collectors’ feedback
	Effective supervision
	Use advisory committees (task force)

Restructuring of the nurses’ role as trauma registrars allowed for 100% patient capture with >90% accuracy and data completeness. This ensured that TR responsibilities are not a hindrance for trauma care providers in a busy clinical environment [31]. However, this requires a new cadre of dedicated trauma data staff, with long-term investment in human resources. The fidelity of the implementation demonstrated that the entire process was well structured and thoroughly planned, and did not require changes. Constant supervision and prompt responses to queries reduced the chances of errors and promoted an environment of effective feedback through centralized technical assistance. The task force conducted regular meetings to share data and feedback, and to keep track of the progress of implementation. The next step is to scale up and integrate the tool with Al-Shifa; with newer mHealth platforms such as kobotoolbox and kobocollect, the tool and database could have more flexibility [32].

The early impact of the IIRU-TR on measuring the burden and in-hospital care processes is encouraging and could build the foundation for a structured quality improvement program [6,10]. Our study shows that a trauma database developed with tablet-based tools in major trauma centers could provide the basis for national injury surveillance and performance improvement initiatives in countries where such systems are underdeveloped and are widely needed [30,33–35]. Scaling up IIRU-TR to inform healthcare providers and administrators with respect to performance improvement and injury prevention is the ultimate goal of this multicenter registry.

## Conclusion

The initial results show the promising potential of a user-friendly, comprehensive electronic TR through the use of mHealth tools. The pilot test in two hospitals indicates that the registry can be used to create a multicenter trauma database. The use of an IT implementation framework helps in better planning and tracking of the implementation process.

## Paper context

Oman is a high-income country with a high burden of injuries. The trauma system is in a developing stage and in-depth data on trauma patients are lacking. This paper provides step-by-step details of the planning, development and implementation of an mHealth-based trauma registry using an implementation framework derived from the Precede–Proceed model of behavioral change. It outlines strategies and factors for a successful implementation of mHealth-based registries.

## References

[1] Wang H, Dwyer-Lindgren L, Lofgren KT, et al Age-specific and sex-specific mortality in 187 countries, 1970–2010: a systematic analysis for the Global Burden of Disease Study 2010. Lancet. 2013;380:2071–9. 10.1016/S0140-6736(12)61719-X 23245603

[2] Mokdad AH, Jaber S, Aziz MIA, et al The state of health in the Arab world, 1990–2010: an analysis of the burden of diseases, injuries, and risk factors. Lancet. 2014;383:309–320. 24452042 10.1016/S0140-6736(13)62189-3

[3] Annual Health Report Department of Health Information and Statistics MoH, (ed.). Muscat, Oman: Ministry of Health, Sultanate of Oman; 2014.

[4] Nwomeh BC, Lowell W, Kable R, et al History and development of trauma registry: lessons from developed to developing countries. World J Emerg Surg. 2006;1:32. 17076896 10.1186/1749-7922-1-32 PMC1635421

[5] O’Reilly GM, Cameron PA, Joshipura M. Global trauma registry mapping: a scoping review. Injury. 2012;43:1148–1153. 22483995 10.1016/j.injury.2012.03.003

[6] Moore L, Clark DE The value of trauma registries. Injury. 2008;39:686–695. 18511052 10.1016/j.injury.2008.02.023

[7] Statistical Year Book Information NCfSa, (ed.). Muscat Oman: National Center for Statistics and Information, Sultanate of Oman; 2016.

[8] Bener A, Abu-Zidan FM, Bensiali AK, et al Strategy to improve road safety in developing countries. Saudi Med J. 2003;24:603–608. 12847587

[9] Mock C, Joshipura M, Arreola-Risa C, et al An estimate of the number of lives that could be saved through improvements in trauma care globally. World J Surg. 2012;36:959–963. 22419411 10.1007/s00268-012-1459-6

[10] Rutledge R The goals, development, and use of trauma registries and trauma data sources in decision making in injury. Surg Clin North Am. 1995;75:305–326. 7900000 10.1016/s0039-6109(16)46590-4

[11] Zehtabchi S, Nishijima DK, McKay MP, et al Trauma registries: history, logistics, limitations, and contributions to emergency medicine research. Acad Emerg Med. 2011;18:637–643. 21676063 10.1111/j.1553-2712.2011.01083.x

[12] Mock C, Juillard C, Brundage S, et al Guidelines for trauma quality improvement programmes. Geneva, Switzerland: World Health Organization Press; 2009 Available from: http://apps.who.int/iris/bitstream/10665/44061/1/9789241597746_eng.pdf.

[13] Mehmood A, Razzak JA, Kabir S, et al Development and pilot implementation of a locally developed Trauma Registry: lessons learnt in a low-income country. BMC Emerg Med. 2013;13:4. 23517344 10.1186/1471-227X-13-4 PMC3606628

[14] Mehmood A, Allen KA, Al-Maniri A, et al Trauma care in Oman: a call for action. Surgery. 2017; pii: S0039-6060(17)30094-6. 10.1016/j.surg.2017.01.028 28351526

[15] Kahn JG, Yang JS, Kahn JS ‘Mobile’ health needs and opportunities in developing countries. Health Affairs. 2010;29:252–258. 20348069 10.1377/hlthaff.2009.0965

[16] Agarwal S, Labrique A Newborn health on the line: the potential mHealth applications. JAMA. 2014;312:229–230. 24953141 10.1001/jama.2014.6371

[17] Labrique AB, Vasudevan L, Kochi E, et al mHealth innovations as health system strengthening tools: 12 common applications and a visual framework. Global Health. 2013;1:160–171. 10.9745/GHSP-D-13-00031 PMC4168567 25276529

[18] Kukafka R, Johnson SB, Linfante A, et al Grounding a new information technology implementation framework in behavioral science: a systematic analysis of the literature on IT use. J Biomed Inform. 2003;36:218–227. 14615230 10.1016/j.jbi.2003.09.002

[19] Gielen AC, McDonald EM, Gary TL, et al Using the precede-proceed model to apply health behavior theories. Health Behav Health Educ. 2008;4:407–429.

[20] Green L, Kreuter M The precede–proceed model In: Health promotion planning: an educational approach. 3rd ed. Mountain View (CA): Mayfield Publishing; 1999 p. 32–43.

[21] Green LW, Kreuter MW Health program planning: an educational and ecological approach. NewYork (NY): McGraw-Hill Companies; 2005.

[22] Holder Y, Peden M, Krug E, et al Injury surveillance guidelines. Geneva: World Health Organization; 2001.

[23] Rotondo MFC, Chris C, Smith RS Resources for optimal care of the injured patient. Chicago, IL: American College of Surgeons; 2014 p. 107.

[24] Baker SP, Oʼneill B, Haddon W Jr, et al The injury severity score: a method for describing patients with multiple injuries and evaluating emergency care. J Trauma Acute Care Surg. 1974;14:187–196. 4814394

[25] Champion HR, Sacco WJ, Copes WS, et al A revision of the trauma score. J Trauma Acute Care Surg. 1989;29:623–629. 10.1097/00005373-198905000-00017 2657085

[26] Osler T, Nelson LS, Bedrick EJ Injury severity scoring. J Intensive Care Med. 1999;14:9–19.

[27] mHealth: New horizons for health through mobile technologies Global observatory for eHealth. Switzerland: World Health Organization; 2011.

[28] Bunger AC, Powell BJ, Robertson HA, et al Tracking implementation strategies: a description of a practical approach and early findings. Health Res Policy Syst. 2017;15:15. 28231801 10.1186/s12961-017-0175-y PMC5324332

[29] Mehmood A, Razzak JA Trauma registry—needs and challenges in developing countries. J Pak Med Assoc. 2009;59:807–808. 20201167

[30] Zargaran E, Schuurman N, Nicol AJ, et al The electronic Trauma Health Record: design and usability of a novel tablet-based tool for trauma care and injury surveillance in low resource settings. J Am Coll Surg. 2014;218:41–50. 24355875 10.1016/j.jamcollsurg.2013.10.001

[31] Prasad MV, Agrawal A, Kumar SS, et al Converting a paper proforma template to a user friendly electronic database to collect traumatic brain injury data. Rom Neurosurg. 2014;21:435–445.

[32] Johnson W, Lin Y, Mukhopadhyay S, Meara J, editors. Surgical care systems strengthening: developing national surgical, obstetric and anaesthesia plans. Geneva (Switzerland): World Health Organization; 2017 Available from: http://apps.who.int/iris/bitstream/10665/255566/1/9789241512244-eng.pdf

[33] Agrawal A Injury surveillance or trauma registry: need of hour and time to start. The Indian Journal of Neurotrauma. 2011;8:37–39.

[34] Schuurman N, Cinnamon J, Matzopoulos R, et al Collecting injury surveillance data in low- and middle-income countries: the Cape Town Trauma Registry pilot. Glob Public Health. 2011;6:874–889. 20938854 10.1080/17441692.2010.516268

[35] O’Reilly GM, Joshipura M, Cameron PA, et al Trauma registries in developing countries: a review of the published experience. Injury. 2013;44:713–721. 23473265 10.1016/j.injury.2013.02.003

